# Sequence variations of the *EGR4* gene in Korean men with spermatogenesis impairment

**DOI:** 10.1186/s12881-017-0408-5

**Published:** 2017-05-02

**Authors:** Se Ra Sung, Seung Hun Song, Kyung Min Kang, Ji Eun Park, Yeo Jung Nam, Yun-jeong Shin, Dong Hyun Cha, Ju Tae Seo, Tae Ki Yoon, Sung Han Shim

**Affiliations:** 1 Genetics Laboratory, Fertility Center of CHA Gangnam Medical Center, Seoul, South Korea; 2 Department of Urology, CHA Gangnam Medical Center, Seoul, South Korea; 3 Department of Urology, Cheil General Hospital, Seoul, South Korea; 4 Department of Obstetrics and Gynecology, CHA Gangnam Medical Center, Seoul, South Korea; 5 Department of Biomedical Science, College of Life Science, CHA University, Seoul, South Korea

**Keywords:** *EGR4* gene, Sequence variation, Impaired spermatogenesis, Male infertility

## Abstract

**Background:**

*Egr4* is expressed in primary and secondary spermatocytes in adult mouse testes and has a crucial role in regulating germ cell maturation. The functional loss of *Egr4* blocks spermatogenesis, significantly reducing the number of spermatozoa that are produced. In this study, we examined whether *EGR4* variants are present in Korean men with impaired spermatogenesis.

**Methods:**

A total 170 Korean men with impaired spermatogenesis and 272 normal controls were screened. The coding regions including exon-intron boundaries of *EGR4* were sequenced by PCR-direct sequencing method.

**Results:**

We identified eight sequence variations in the coding region and 3′-UTR regions of the *EGR4* gene. Four were nonsynonymous variants (rs771189047, rs561568849, rs763487015, and rs546250227), three were synonymous variants (rs115948271, rs528939702, and rs7558708), and one variant (rs2229294) was localized in the 3′-UTR. Three nonsynonymous variants [c.65_66InsG (p. Cys23Leufs*37), c.236C > T (p. Pro79Leu), c.1294G > T (p. Val432Leu)] and one synonymous variant [c.1230G > A (p. Thr410)] were not detected in controls. To evaluate the pathogenic effects of nonsynonymous variants, we used seven prediction methods. The c.214C > A (p. Arg72Ser) and c.236C > T (p. Pro79Leu) variants were predicted as “damaging” by SIFT and SNAP^2^. The c.65_66insG (p. Cys23Leufs*37) variants were predicted as “disease causing” by Mutation Taster, SNPs &GO and SNAP^2^. The c.867C > G (p. Leu289) variants were predicted as “disease causing” only by Mutation Taster.

**Conclusion:**

To date, this study is the first to screen the *EGR4* gene in relation to male infertility. However, our findings did not clearly explain how nonsynonymous *EGR4* variations affect spermatogenesis. Therefore, further studies are required to validate the functional impact of *EGR4* variations on spermatogenesis.

**Electronic supplementary material:**

The online version of this article (doi:10.1186/s12881-017-0408-5) contains supplementary material, which is available to authorized users.

## Background

The early growth response (EGR; MIM# 128992) proteins are a family of zinc finger transcription factors that moderate the regulation of gene expression in response to receptor ligand binding [1]. The *EGR* family consists of *EGR1* (NGFI-A), *EGR2* (Krox20), *EGR3*, and *EGR4* (NGFI-C, pAT133) [2, 3]. The zinc finger motifs bind to a specific 9 base pair (bp) consensus sequence (−GCGGGGGCG-) within the promoter regions of downstream genes for transcriptional activation [4, 5]. *Egr* knock-out mice have provided insights into the biological functions of Egr. For example, Egr1 regulates luteinizing hormone (LH) β expression, and female *Egr1-*knockout mice are infertile [6]. *Egr2-* and *Egr3*-null mice also have specific abnormalities. Moreover, human *EGR2* mutations have been identified in patients with congenital hypomyelinating neuropathy or type 1 day Charcot-Marie-Tooth disease [7].

*Egr4* is known to be ubiquitously expressed in the central nervous system. However, Tourtellotte et al. found low levels of *EGR4* expression in maturing male germ cells. *Egr4* expression was detected in primary and secondary spermatocytes in adult mouse testes and had a crucial role in spermatogenesis by regulating germ cell maturation during early-mid pachytene. The functional loss of *Egr4* blocked spermatogenesis, leading to a significant reduction in spermatozoa production [8]. In another study, Hogarth et al. also reported *Egr4* expression in murine testes and suggested that it may regulate multiple stages of spermatogenesis [9].

To date, only a few studies have investigated the function of the *EGR4* gene in humans. Matsuo et al. suggested that EGR4 may regulate bone metastasis and the proliferation of small cell lung cancer cells [10]. EGR4 also regulates the secretion of LH and has a role in the fertility of cryptorchidism patients [11]. However, the relationship between EGR4 and impaired spermatogenesis has not been studied. In this study, we examined for the first time whether sequence variations in the *EGR4* gene are present in men with idiopathic non-obstructive azoospermia.

## Methods

### Subjects

A total of 170 Korean men with impaired spermatogenesis [51 with oligozoospermia aged 33.9 ± 5.00 (age in years, ± standard deviation)) and 119 with azoospermia (aged 34.3 years ± 5.28)] and 272 normal controls (aged 34.5 years ± 4.61) were recruited from the CHA Gangnam Medical Center at CHA University between January 2010 and December 2012. Patients with tubule obstruction, chromosome abnormality, or a microdeletion in the AZF region of the Y chromosome were excluded. Normal controls had a normal sperm count and no history of infertility. Semen was analyzed according to the 1999 World Health Organization criteria.

### DNA extraction

Genomic DNA was extracted from peripheral blood samples using the QuickGene DNA blood kit (Fujifilm, Japan) according to the manufacturer’s instructions. DNA yield was quantified using a NanoDrop™ spectrophotometer (Thermo Scientific, Maryland, USA). Extracted DNA was stored at−80 °C until further analysis.

### Sequence analysis of *EGR4*

**Fig. 1:**
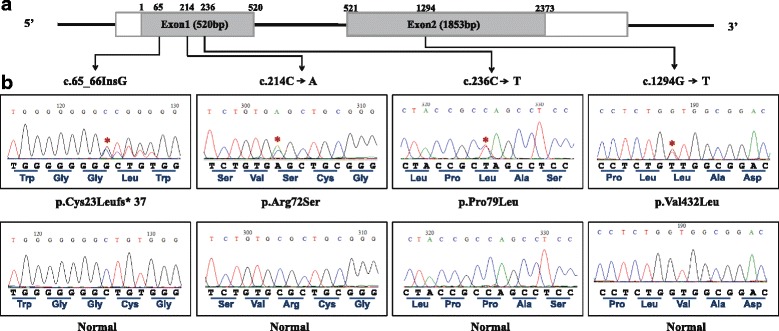
Identification of variants in the *EGR4* gene. **a** Schematic representation of the *EGR4* locus containing the two exons that were sequenced. Coding regions are indicated by a *black* box. Four nonsynonymous variations (c.65_66InsG, c.214C > A, c.236C > T, and c.1294G > T) identified in Korean patients with oligo/azoospermia are illustrated. **b** Electropherograms showing the variant sequences. Variants identified in the azoo-/oligozoospermia group (c.65_66InsG, c.214C > A, c.236C > T, and c.1294G > T) are shown in the upper panel. Wild-type sequences from the control group are shown in the lower panel. *Red* stars indicate the variant locations

The coding regions of *EGR4* were screened by PCR and direct sequencing. PCR primers for two exons and their intron boundaries were designed using Primer 3 (http://bioinfo.ut.ee/primer3-0.4.0/). The locations and sequences of primer sets are presented in Fig. 1 and Table 1. Because of their large size, the exons were divided: exon 1 was divided into two and exon 2 was divided into six overlapping fragments. The GC-rich PCR system (Roche Diagnostics, Mannheim, Germany) was used for exon 1, and the Hotstart Taq PCR premix (Bioneer, Daejeon, Republic of Korea) was used for exon 2. Thermal cycling conditions were as follows: initial denaturation at 94 °C for 5 min, 30–35 cycles for 30 s at 94 °C, 30 s at 60 °C, and 30 s at 72 °C, with a final extension at 72 °C for 10 min. PCR products were loaded on a 2% agarose gel and then purified with ExoSAP-IT (USB Corporation, Cleveland, OH). The sequencing reaction was performed using BigDye® Terminator v3.1 Cycle Sequencing Kit (Applied Biosystems, Austin, TX) according to the manufacturer’s instructions. After the sequencing reaction, 55 μl of BigDye® X-Terminator™ (Applied Biosystems, Bedford, MA) solution was added directly to the sequencing reaction plate well and vortexed for 30 min at 1800 rpm. After vortexing, the reaction plate was briefly centrifuged, and the supernatant was loaded onto an ABI 3130XL Genetic Analyzer using the BigDye® X-Terminator run module. All sequence reactions were performed in forward and reverse directions to eliminate error.

**Table 1:** Primer sequences

No.	Exon No.	Genome Location	Size		Sequence (5′ → 3′)
1	Exon1-1	Chr2:73293714	423 bp	F	GAGCTTTCCTTTTCGGGAGT
		−73293292		R	TCGGAAAACTCGCTAAGGTG
2	Exon1-2	Chr2:73293535	448 bp	F	CTTTGGAGAGGCGAGGAG
		−73293088		R	TAGCTCCAATGTCCCAGTCC
3	Exon2-1	Chr2: 73292868	398 bp	F	GTGGGCACCAAGAGTTTTGT
		−73292471		R	CAGATCCGGGGAGTAAAGGT
4	Exon2-2	Chr2: 73292610	434 bp	F	ACCTCATGTCGGGCATCTTA
		−73292177		R	GCTGATGGACAGCAAGTCCT
5	Exon2-3	Chr2: 73292415	433 bp	F	CCTCGCAGTGCCTGTATGAG
		−73291983		R	GAAAGCTGAGGCTGCGTACT
6	Exon2-4	Chr2: 73292154	410 bp	F	TCCCAGCCAACAGACTCTATC
		−73291745		R	AAAGCTCCGCACACAACTCT
7	Exon2-5	Chr2: 73291928	382 bp	F	GGACATCCCTGGAAGCAGT
		−73291547		R	GCTGTGCCGTTTCTTCTCAT
8	Exon2-6	Chr2: 73291764	401 bp	F	AGAGTTGTGTGCGGAGCTTT
		−73291364		R	CGAGGAGGAGTTGGAAGAAG

### Statistical analysis and database search

For each sequence variation, data were statistically analyzed using Statistical Package for Social Sciences (SPSS) version 22 software (Chicago, IL, USA). To evaluate the association between patient and control groups, the odds ratio (OR), 95% confidence interval (CI) and applied *p* values were calculated using the chi-square test and Fisher’s exact test (two-tailed). An applied *p* value of less than 0.05 was considered statistically significant. SIFT [12, 13], PolyPhen-2 [14, 15], Mutation Taster [16, 17], fathmm [18], Mutation assessor [19], SNPs &GO [20, 21] and SNAP^2^ [22] databases were used to predict potentially damaging effects of the identified sequence variations.

## Results

We identified eight sequence variations in the coding region and 3′-UTR of the *EGR4* gene in our Korean population. The locations, types, and allele frequencies of the variations are presented in Fig. 1 and Table 2. Four were nonsynonymous variants (rs771189047, rs561568849, rs763487015, and rs546250227), three were synonymous variants (rs115948271, rs528939702, and rs7558708), and one (rs2229294) was localized in the 3′-UTR. Three nonsynonymous variants [c.65_66InsG (p. Cys23Leufs*37), c.236C > T (p. Pro79Leu), c.1294G > T (p. Val432Leu)] and one synonymous variant in exon 2 (c.1230G > A) were detected only in patients (Fig. 1). The c.214C > A (p. Arg72Ser) and c.867C > G (p. Leu289) variants were identified in both patients and controls. The genotype frequencies of *EGR4* c.65_66InsG**,** c.214C > A, c.236C > T**,** c.867C > G, c.1230G > A, c.1294G > T, and c.1488C > T variants were not significantly different between the patient and the control groups (Table 3). We evaluated the pathogenic effects of the nonsynonymous variants using 7 programs by PolyPhen-2, SIFT, Mutation Taster, fathmm, Mutation assessor, SNPs &GO and SNAP^2^ (Additional file 1: Table S1). The c.214C > A (p. Arg72Ser) and c.236C > T Pro79Leu) variants were predicted as “damaging” by SIFT and SNAP^2^. The c.65_66insG (p. Cys23Leufs*37) variants were predicted as “disease causing” by Mutation Taster, SNPs &GO and SNAP^2^. The c.867C > G (p. Leu289) variants were predicted as “disease causing” by only Mutation Taster.

**Table 2:** *EGR4* sequencing results from the non-obstructive azoospermia group

				Allele Frequency (%)					
				Oligozoospermia/Azoospermia			Normal Group		
				(*n* = 170)			(*N* = 272)		
Location	Variation	Amino Acid Variation	dbSNP ID	Wild Type	Heterozygote	Homozygote	Wild Type	Heterozygote	Homozygote
Exon 1	c.65_66InsG	p. Cys23Leufs*37	rs771189047	169 (99.41)	1 (0.59)	0	272 (100)	0	0
Exon 1	c.214C > A	p. Arg72Ser	rs561568849	168 (98.88)	2 (1.18)	0	270 (92.26)	2 (0.74)	0
Exon 1	c.236C > T	p. Pro79Leu	rs763487015	169 (99.41)	1 (0.59)	0	272 (100)	0	0
Exon 2	c.867C > G	p. Leu289	rs115948271	167 (98.24)	3 (1.76)	0	265 (97.42)	7 (2.58)	0
Exon 2	c.1230G > A	p. Thr410	rs528939702	169 (99.41)	1 (0.59)	0	245 (100)	0	0
Exon 2	c.1294G > T	p. Val432Leu	rs546250227	169 (99.41)	1 (0.59)	0	245 (100)	0	0
Exon 2	c.1488C > T	p. Arg496	rs7558708	40 (23.53)	95 (55.88)	35 (20.59)	70 (25.74)	141 (51.84)	61 (22.42)
3′-UTR	c.2373 + 52C > T	Non-coding	rs2229294	40 (23.53)	95 (55.88)	35 (20.59)	70 (25.74)	141 (51.84)	61 (22.42)

**Table 3:** Genotypes and allele distributions of *EGR4* SNPs

dbSNP ID	Variation	Genotype	Cases (%)	Control (%)	Odds Ratio	95% CI	*P* Value
rs771189047	c.65_66InsG	−/−	169 (99.4)	272 (100)	1.00		
		-/G	1 (0.6)	0 (0)	2.609	2.318–2.937	0.385
		G/G	0 (0)	0 (0)	-	-	-
		**G allele**	**1**	**0**	**2.609**	**2.318–2.937**	**0.385**
rs561568849	c.214C > A	C/C	168 (98.8)	270 (99.3)	1.00		-
		C/A	2 (1.2)	2 (0.7)	1.607	0.224–11.517	0.641
		A/A	0 (0)	0 (0)	-	-	-
		**A allele**	**2**	**2**	**1.607**	**0.224–11.517**	**0.641**
rs763487015	c.236C > T	C/C	169 (99.4)	272 (100)	1.00		-
		C/T	1 (0.6)	0 (0)	2.609	2.318–2.937	0.385
		T/T	0 (0)	0 (0)	-	-	-
		**T allele**	**1**	**0**	**2.609**	**2.318–2.937**	**0.385**
rs115948271	c.867C > G	C/C	167 (98.2)	265 (97.4)	1.00		-
		C/G	3 (1.8)	7 (2.6)	0.680	0.173–2.666	0.746
		G/G	0 (0)	0 (0)	-	-	-
		**G allele**	**3**	**7**	**0.680**	**0.173–2.666**	**0.746**
rs528939702	c.1230G > A	G/G	169 (99.4)	272 (100)	1.00		
		G/A	1 (0.6)	0 (0)	2.609	2.318–2.937	0.385
		A/A	0 (0)	0 (0)	-	-	-
		**A allele**	**1**	**0**	**2.609**	**2.318–2.937**	**0.385**
rs763487015	c.1294G > T	G/G	169 (99.4)	272 (100)	1.00		-
		G/T	1 (0.6)	0 (0)	2.609	2.318–2.937	0.385
		T/T	0 (0)	0 (0)	-	-	-
		**T allele**	**1**	**0**	**2.609**	**2.318–2.937**	**0.385**
rs7558708	c.1488C > T	C/C	40 (23.5)	70 (25.7)	1.00		
		C/T	95 (55.9)	141 (51.8)	0.848	0.531–1.354	0.554
		T/T	35 (20.6)	61 (22.4)	0.996	0.564–1.759	1.000
		**T allele**	**165**	**263**	**0.993**	**0.757–1.302**	**1.000**

## Discussion

In this study, we identified sequence variations in the *EGR4* gene of patients with idiopathic non-obstructive spermatogenetic impairment. Spermatogenesis is followed by the differentiation of primordial germ cells into motile spermatozoa. This process is controlled by numerous factors [23, 24], and disruption of these factors may affect the quality and quantity of spermatozoa production and fertility in males. Many genes have been associated with spermatogenesis, but the pathophysiological mechanisms of these genes have not been elucidated. *EGR4* is expressed in germ cells and has a crucial role in spermatogenesis by regulating germ cell maturation during pachytene. In addition, the functional loss of *EGR4* blocks spermatogenesis, thereby reducing spermatozoa production [8].

We identified eight variants in the *EGR4* gene of Korean males with impaired spermatogenesis. However, these variants were all listed in the NCBI Single Nucleotide Polymorphisms (SNP) database [25], and the allele frequencies of the variants alleles were not significantly different between patients and controls. Therefore, the significance of these variations in spermatogenesis is not clear. The allele frequency of each variant was very low (less than 0.01), and variants not found in the controls were only identified in one patient. We were also unable to elucidate the biochemical and physiological significance of each variant. Instead, we evaluated the pathological significance of the variants using 7 computational prediction algorithms: PolyPhen-2, SIFT, Mutation Taster, fathmm, Mutation assessor, SNPs &GO and SNAP^2^.

Four variants were not identified in the controls, and three were nonsynonymous. The c.65_66InsG (p. Cys23Leufs*37) variant was detected in only one patient with oligozoospermia. This insertion shifted the reading frame and generated a premature stop codon. It was predicted as “damaging” by the Mutation Taster and SNAP2 database. Haploinsufficiency is a well-known mechanism of many genetic diseases [26–28]. In null mouse models, hemizygotes (+/−) may show a normal phenotype because a deleted allele may not affect its phenotype. However, a gene product of a variant allele may interfere with a normal gene product. The allele frequency of the insertion was very rare and may have been responsible for the reduced sperm count in the patient.

The c.236C > T (p. Pro79Leu) variant was detected in only one patient. The variant was also identified by the 1000 genomes project with a MAF of 0.0003. Proline and leucine are nonpolar amino acids, so the mutability of this variant should be negligible. However, this type of substitution could affect protein function. Computational analyses predicted the variant as both “damaging” (SIFT) and “benign” (PolyPhen-2); therefore, its pathological significance is unclear. The nonsynonymous variant c.1294G > T (p. Val432Leu) was also only identified in one patient and had already been identified by the 1000 genomes project with a MAF of 0.0016. Valine and leucine are structurally similar; therefore, nonpolar amino acids of this variant should be insignificant. The synonymous c.1230G > A (p. Thr410) variant was also only found in one patient with impaired spermatogenesis and had also previously been identified by the 1000 genomes project with a MAF of 0.0008.

The remaining four variants, including one nonsynonymous variant, were identified in both patients and controls. The c.214C > A (p. Arg72Ser) variant had a MAF of 0.009, and the c.867C > G (p. Leu289) variant had a MAF of 0.023 in our Korean population. These frequencies were much higher than those reported in the NCBI SNP database. The c.867C > G (p. Leu289) variant was predicted to be “disease causing” by Mutation Taster. The substitution may affect a splice site [16, 17]. However, this variant occurred more frequently in controls and is considered to be a normal variation in the Korean population. Two of our identified variants (c.1488C > T and c2373 + 52C > T) are considered common variants; c.1488C > T has been reported in European (MAF: 0.0636) and African (MAF: 0.4554) populations and was also identified by the 1000 genomes project (MAF: 0.2845). Here, we found that the MAF for this variant in a Korean population is similar to that previously reported in an African population. The c.2373 + 52C > T variant was located in the 3′-UTR and was tightly linked to the c.1448C > T variant in our Korean population; the C allele of the c1448C > T variant was coupled to the C allele of the c.2373 + 52C > T variant. The haplotype frequencies did not differ between the two groups.

SNPs and other structural variants have been associated with impaired spermatogenesis in different populations, but the same variants have not yet been reported in more than one population [29–32]. Chuncheng et al. performed the largest genetic association study in patients with non-obstructive azoospermia and identified SNPs associated with five potentially related genes. They suggested that these SNPs act as cofactors rather than directly affecting spermatogenesis because they were also present at a lower frequency in fertile men. These variants may cause mild impairment of spermatogenesis, but this could be worsened in the presence of other cofactors [33]. Tourtellotte et al. suggested that the *EGR4* gene can compensate for the function of *EGR1* in regulating LH during steroidogenesis [34]. This supports the idea that *EGR1* has a dominant role in maintaining male fertility, while *EGR4* can compensate for the functional loss of *EGR1* in germ cells.

## Conclusions

Eight variations were detected in the *EGR4* gene of Korean men with idiopathic spermatogenetic impairment. To the best of our knowledge, this is the first screening of the *EGR4* gene in relation to male infertility. Our findings did not fully elucidate how the identified variants affect spermatogenesis.

Our results found no difference in mutation frequency between cases and controls, and there is no evidence that heterozygous EGR4 variations are associated with infertility in humans.

Nevertheless, further studies are required to validate whether these variants affect *EGR4* gene function and increase the risk of male infertility associated with other genetic changes, such as *EGR1* mutations.

## Additional file

Additional file 1: Table S1. Summary of variants identified in the *EGR4* gene. (DOCX 16 kb)
