# Supplementary material for: Sequence variations of the EGR4 gene in Korean men with spermatogenesis impairment
Source: BMC Med Genet. 2017 May 2;18:47. doi: 10.1186/s12881-017-0408-5 (PMC5414287; doi:10.1186/s12881-017-0408-5)
Supplement: Additional file 1: Table S1. — Summary of variants identified in the EGR4 gene. (DOCX 16 kb) [file 12881_2017_408_MOESM1_ESM.docx]

**Additional file 1: Table S1. Summary of variants identified in the *EGR4* gene**

|  | | | | In silico Novel Variant Analysis | | | | | | |
| --- | --- | --- | --- | --- | --- | --- | --- | --- | --- | --- |
| Location | **Variation** | **Amino acid variation** | **dbSNP ID** | **PolyPhen-2^†^** | **SIFT^††^** | **Mutation Taster^†††^** | **fathmm^‡^** | **Mutation**  **assessor^‡‡^** | **SNPs &GO^‡‡‡^** | **SNAP2^‡‡‡‡^** |
| Exon 1 | c.65_66InsG | p.Cys23Leufs*37 | rs771189047 | NA | NA | Disease causing | Tolerated | neutral | Disease | Effect |
| Exon 1 | c.214C>A | p.Arg72Ser | rs561568849 | Benign | Damaging | Polymorphism | Tolerated | neutral | Neutral | Effect |
| Exon 1 | c.236C>T | p.Pro79Leu | rs763487015 | Benign | Damaging | Polymorphism | Tolerated | neutral | Neutral | Effect |
| Exon 2 | c.867C>G | p.Leu289 | rs115948271 | Benign | Tolerated | Disease causing | NA | NA | NA | Neutral |
| Exon 2 | c.1230G>A | p.Thr410 | rs528939702 | Benign | Tolerated | Polymorphism | NA | NA | NA | Neutral |
| Exon 2 | c.1294G>T | p.Val432Leu | rs546250227 | Benign | Tolerated | Polymorphism | Tolerated | low | Neutral | Neutral |
| Exon 2 | c.1488C>T | p.Arg496 | rs7558708 | Benign | Tolerated | Polymorphism | NA | NA | NA | Neutral |

^†^; http://genetics.bwh.harvard.edu/pph2/

^††^; http://siftdna.org/

^†††^; www.mutationtaster.org

**^‡^**; fathmm.biocompute.org.uk

**^‡‡^;** mutationassessor.org

**^‡‡‡^; s**nps.biofold.org/snps-and-go

**^‡‡‡‡^;** https://rostlab.org/services/SNAP/
